# Transmission dynamics of pulmonary tuberculosis between autochthonous and immigrant sub-populations

**DOI:** 10.1186/1471-2334-9-197

**Published:** 2009-12-04

**Authors:** Judit Barniol, Stefan Niemann, Valérie R Louis, Bonita Brodhun, Caroline Dreweck, Elvira Richter, Heiko Becher, Walter Haas, Thomas Junghanss

**Affiliations:** 1 Section Clinical Tropical Medicine, Department for Infectious Diseases, University Hospital Heidelberg, Im Neuenheimer Feld 324, 69120 Heidelberg, Germany; 2 Institute of Public Health, University Hospital Heidelberg, Im Neuenheimer Feld 324, 69120 Heidelberg, Germany; 3 National Reference Centre for Mycobacteria, Research Centre Borstel, Parkallee 18, 23845 Borstel, Germany; 4 Department for Infectious Disease Epidemiology, Robert Koch Institute, DGZ-Ring 1, 13086 Berlin, Germany; 5 State Health Office Baden-Württemberg (Landesgesundheitsamt), Postfach 10 29 42, 70025 Stuttgart, Germany

## Abstract

**Background:**

The overall incidence of tuberculosis (TB) in Western Europe has been declining since the 19^th ^Century. However, immigrant sub-groups from high-prevalence countries are slowing down this trend. The aim of this study was to describe how immigration influences TB transmission in Germany. For that we prospectively investigated the dynamics of TB transmission between TB high-prevalence immigrant and TB low-prevalence local populations with molecular epidemiological methods and conventional contact investigations. Besides, we assessed transmission in relation to social mixing using an innovative tool that measures the integration of immigrants into the local social environment.

**Methods:**

A prospective study of confirmed culture positive cases of pulmonary TB and their contacts was carried out in a German federal state from 2003 to 2005. Data for the study included: 1) case data routinely collected by the local public health staff and transmitted to the state health office and the national surveillance centre, 2) a study questionnaire designed to capture social interactions of relevance for TB transmission and 3) molecular genotyping data (IS*6110 *DNA fingerprint and spoligotyping). The proportion of German cases caused by foreign-born cases, and vice versa, was estimated and an integration index was computed using a selected set of questions from the study questionnaire.

**Results:**

A total of 749 cases of culture-positive pulmonary tuberculosis voluntarily enrolled in the study, representing 57.8% of all registered cases diagnosed over the study period. Data that included study questionnaire and DNA fingerprinting were available for 41% (n = 308) of the study participants. Forty-seven clusters, defined as a least two cases infected by the same TB strains, were identified by molecular methods and included 132 (17%) of the study participants. Epidemiological links were identified for 28% of the clusters by conventional epidemiological data. In mixed clusters, defined as clusters including German and foreign-born individuals, the probability of cases to be caused by foreign-born cases was estimated at 18.3%. We observed a trend to mixed clusters with increasing time spent by immigrants in the host country. This group also presented comparatively higher integration indexes than immigrants in immigrant-only clusters.

**Conclusion:**

Our results confirm the findings of other studies that there is no significant TB transmission from TB high-prevalence immigrant to TB low-prevalence autochthonous population. This may be explained by the good performance of tuberculosis screening programmes for certain groups arriving in Germany from high- prevalence countries, by a low degree of mixing of immigrants with the local population or by a combination of both.

## Background

Although the incidence of TB in Western Europe has been steadily declining in the last decades, immigrants coming from high-endemic areas are slowing down this trend. There are 7.3 million foreigners living in Germany, 8.9% of the total population. The incidence of tuberculosis in foreign citizens was 27.4 per 100,000 in 2005. This was 5.4 times higher than in Germans [1]. The impact on the epidemiology and the transmission patterns of TB need to be captured and understood to adapt TB control strategies. Deoxyribonucleic Acid (DNA) fingerprinting of *Mycobacterium tuberculosis *(MTB) provides a powerful tool to trace and identify TB transmission chains and outbreaks. Linkages of this information to unique features of sub-populations including social behaviour and integration patterns will facilitate the identification of chains of transmission and, ultimately, the design and implementation of appropriate control strategies.

Our study objectives were to prospectively describe the dynamics of TB transmission between immigrant and local populations in one of the federal states of Germany (Baden-Württemberg) with the help of molecular epidemiological tools and traditional contact investigations and to assess transmission in relation to the degree of integration. For this purpose we used a new tool to measure integration among sub-groups with the help of an integration index, which was computed based on a selected set of answers to the study questionnaire about socio-economic, social behaviour and integration issues.

Several studies have already addressed the issue of TB transmission between populations from high- and low-prevalence countries in different countries. Most of them are retrospective molecular epidemiological studies [2-7] or studies combining DNA fingerprinting with traditional contact investigations [8-15]. Generally, no major risk of transmission from immigrants into the autochthonous population has been found.

The proportion of cases in DNA fingerprint clusters in the above studies has ranged from 15.8% to 59% and the proportion of epidemiologically confirmed clusters, for those studies with available data, has remained quite low (10 to 31%).

## Methods

A prospective study of incident confirmed culture-positive cases of pulmonary TB, from this point onwards referred as cases, and their contacts as identified through the public health authorities was carried out in Baden-Württemberg, Germany from 01.01.2003 to 31.12.2005 [16,17].

### Data source

Data for the study were obtained from:

1) case data routinely collected by the local public health staff using standard reports and, once depersonalized, transmitted via the state health office of Baden-Württemberg to the national surveillance centre, Robert Koch Institute (RKI). The data include demographic and clinical data of the patient, laboratory results, Drug Susceptibility Tests (DST), and treatment outcomes (success, failure, death, defaulters) according to the WHO working group recommendations [18].

2) Study questionnaire specifically designed to capture social interactions of relevance for TB transmission. The following variables were assessed: age, gender, nationality, country of birth, year of arrival in Germany, number of visits to the country of birth and length of the visit, relatives/friends still living in the country of birth, postal code in Germany, type of housing, number of rooms, number of people living in the same house/sharing the same room and their nationality, nationality of the neighbours, profession and occupation, nationality of colleagues/classmates, where and with whom spare time is spent, behavioural habits (smoking, eating warm meals, alcohol drinking), known contact to other TB patients, knowledge of German language. See additional file 1: Study questionnaire in English, and

3) molecular genotyping data from the National Reference Centre (NRC). These data contain the following variables: genotype, DNA fingerprint cluster, cluster number, number of bands, spoligotyping results, date of IS*6110 *Restriction Fragment Length Polymorphism analysis (RFLP) (for details see below).

Immigrant TB cases were defined as individuals whose country of birth was not Germany, even if they later acquired German citizenship. Children of immigrants born in Germany were considered Germans. The statistics of immigrants living in Baden-Württemberg were retrieved from the *Statistisches Landesamt Baden-Württemberg *(available at: http://www.statistik-portal.de/BevoelkGebiet/Landesdaten/LRt0601.asp). Figures from the Statistics Bureau are based on nationality of the immigrants and do not include the foreign-born who have acquired German nationality (i.e. German resettlers), thus underestimating the proportion of immigrants.

The tuberculosis screening program for immigrants varies according to the very heterogeneous groups of migrants in Germany. Asylum seekers and some sub-groups of immigrants (e.g. German resettlers (in German *Spätaussiedler*) from the Former Soviet Union (FSU) and East Europe) are actively screened for active disease at the time of arrival in Germany [19,20] and treated as needed according to the German social welfare Law for asylum seekers ("*Asylbewerberleistungsgesetz*").

### Genotyping and cluster identification

All cultures available at the NRC were analysed by IS*6110 *DNA fingerprinting (*IS6110*-RFLP) and spoligotyping according to an internationally standardized protocol as described elsewhere [21,22].

The DNA typing patterns were digitized, analysed with the Bionumerics software (version 4.5, Applied Maths, St-Martin-Latem, Belgium) and stored in a database. Subsequently a cluster analysis was performed as follows: IS*6110 *RFLP clusters were defined based on fully identical patterns (the same number of IS*6110 *bands and identical positions [positions tolerance, 1.2%]). DNA fingerprint and spoligotype patterns were reported to the study centre in Heidelberg and to the local health authorities for further epidemiological analysis. Furthermore, based on genotyping results, the strains were classified into main MTBC (*Mycobacterium tuberculosis *complex) genotypes to allow the analysis of possible associations between strains genotype and patient characteristics.

Recent studies have shown that the MTBC strains can be subdivided in several phylogentic lineages that are differentially prevalent in different geographical areas [23,24]. Furthermore, there is growing evidence that strains of the MTBC are genetically more diverse than previously anticipated and that this genetic heterogeneity indeed influences the transmissibility and virulence of clinical isolates [25,26].

Genotype identification was carried out by using the MIRU-VNTR*plus *webpage [27].

Contact investigations were performed routinely on all cases. Once clusters were confirmed using molecular genotyping, public health offices with clustered patients were requested to extend their general contact investigations through systematic review of patient records and re-interviews to verify the duration and nature of possible contacts, etc. (epidemiological contact tracing). Three types of links were established through conventional epidemiological methods: 1) patients with definite epidemiologic links, 2) patients with probable links, or 3) patients with possible links [28].

Anti TB drug resistance was extracted from the case reports and recorded where available. These data were used as additional information to identify clusters with recent transmission.

### Estimation of transmission

To estimate the proportion of German cases caused by immigrant cases only clustered isolates were considered, since no information on transmission was available for the unique isolates. The proportion of German cases caused by foreign-born cases was estimated as follows: We denoted i = 1,..., i be the index for clusters, I the number of clusters, J_i _the number of members in cluster i, j the index for the j-th member in that cluster and finally p_ijk _the probability that cluster member j of cluster i was infected by cluster member k of that cluster such that . For these probabilities we made the following assumptions:

- If from epidemiological tracing the chain of infection is known, then  for that member κ* of the cluster which infected member j and *p*_ijκ _= 0 for all other members

- If the date of diagnosis differs more than 12 months between two individuals within a cluster, then the probability that the individual with the earlier diagnosis was infected by the other is low and for estimation processes set to zero.

- If no epidemiological tracing was possible, the probability p_ijk _was estimated as 1/(number of individuals within the cluster diagnosed up to 12 months before, or after the diagnosis of the respective case).

- The first member of a cluster was assigned no infection probability if no other member of the cluster was diagnosed within 12 months

We denoted p_ijF _be the probability that cluster member j of cluster i was infected by a non-German. It was calculated by  where summation was over all foreigners within a cluster. The proportion of German cases caused by foreign-born cases p_F _was then estimated by  (1) where the summation was over all N German members of the clusters to which an infection probability could be assigned according to the definition. We compared this estimate with the respective probability if there was a random mixing between foreigners and Germans, taking into account the current proportion of TB cases in foreigners and Germans.

We also estimated the corresponding proportion of foreign cases caused by German cases for which the same method applies.

### Calculation of integration index

An integration index for immigrants was calculated based on the standardized score sum of 11 selected variables from the study questionnaire. Missing values were assigned a zero. The integration index was standardized to range from 0 to 10 by a linear transformation. For a list of questions and scores used for the computation of the integration index see additional file 2: Questions and scoring used for the computation of the integration index.

All analyses were done with the statistic software package SAS and Stata.

### Ethical clearance and informed consent

Ethical clearance for the study was obtained from the ethical committee of the University Hospital Heidelberg. Participation in the study was voluntary and written consent was sought prior to enrolment.

## Results

**Figure 1:**
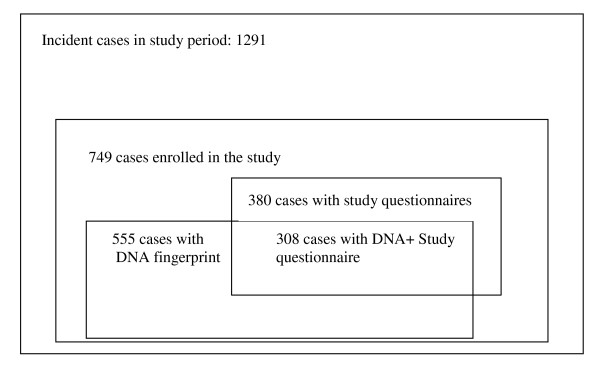
**Study population diagram**. Study population sub-groups and type of data collected.

The study population and sub-groups are displayed in Figure 1: Study population sub-groups and type of data collected. A total of 749 cases of culture-positive pulmonary tuberculosis were enrolled in the study, representing 58% of all cases (n = 1291) diagnosed in Baden-Württemberg over the study period, including 7 cases that belonged to clusters but were diagnosed or reported in late 2002 (2 cases) or early 2006 (5 cases) and who agreed to have their isolates typed. In our study the average incidence rate for the period 2003-2005 was 4.0 per 100.000 of culture positive pulmonary TB.

Study questionnaires were filled in and sent to the study centre for 380 of these 749 cases (51%). For all study participants, national epidemiological surveillance data were available. Epidemiological contact tracing information was collected for 671 contacts.

As of March 2006, isolates for 636 cases were received at the NRC. Removing the cases that were atypical mycobacteria, double-registered, misclassified or for which culture did not grow, and adding the 7 cases belonging to 2002 and 2006, DNA fingerprinting results were available for 555 cases (74% of all culture-positive cases participating in the study).

Complete data (epidemiological data, study questionnaire and results of the DNA fingerprinting) were available for 308 (41%) of the study participants.

The profile of the study participants did not differ from that of the overall cases in Baden-Württemberg in terms of gender (62% males, 37% females), age distribution (mean 49.6 years for males SD: 18.7; mean 47.3 years for females SD: 22.8, nationality and country of birth. Foreign-born study participants were significantly younger than Germans, this being more accentuated in the case of male immigrants (mean 46 years 95% CI 43.8 - 48.3 versus mean 53.7 years 95% CI 51.2- 56.2 for German male. Female immigrants had mean 44 years (95% CI: 40.9 - 47.2) compared to a mean 52.8 years (95% CI: 47.9 - 57.7) for German females.

The proportion of immigrants enrolled in the study was 57.7% (432/749). While 40.7% of the study participants were born in Germany, 57% actually had German nationality. This is due to the fact that an important sub-group of the foreign-born in our study population, the so-called German resettlers, are ethnic Germans born in the FSU (11%) and in East Europe (5%) who, since 1986, have been given the chance to immigrate to Germany and obtain German citizenship. The second largest group of immigrants were those coming from East Europe (10%). Turkish immigrants alone represented 6.4% of the total foreign-born population. The rest (34%) of the immigrants came from 43 different countries. For 12 cases (1.6%) no country of birth was recorded. The proportion of immigrants in Baden-Württemberg per year compared with the proportion of the immigrant TB cases of all TB cases in Baden-Württemberg per year remained constant over the three year study period: immigrants accounted for around 12% of the total population (10.7 million) and for 57% of the total reported cases [29].

### Clusters based on molecular typing data

As summarized in Table 1, of the study participants for which mycobacterial genotyping were available (n = 555), 132 (24%) were classified into 47 DNA fingerprint clusters and the rest (66%) had a unique isolate. Of the 132 clustered isolates, 69 belonged to foreign-born, 60 to native-born Germans and for 3 cases no country of birth was available. Cluster sizes ranged from 2 to 9 persons. There were a total of 28 multinational clusters (composed of cases from 2 to 4 different countries), of which 16 were mixed clusters (with Germans and immigrants) and 12 were immigrant clusters. 21 of the 28 multinational clusters were represented by two countries and those with most representation (4 countries) were mainly composed of immigrants from different FSU countries.

**Table 1:** *M. tuberculosis *complex genotypes of clustered and non-clustered patients in association with country of birth

Genotype	**Numbers of cases not-clust**.	Number of cases in cluster	Range of Cluster	Total	Numbers of clusters by country of birth		
					**All German**	**All Foreign**	**Mixed**

Beijing	23 (5.4%)	26 (19.7%)	2-9	**7**	0	5	2

Haarlem	146 (34.5%)	20 (15.1%)	2-3	**9**	6	1	2

EAI	18 (4.5%)	5 (3.8%)	2	**2**	0	2	0

Delhi	16 (3.8%)	8 (6%)	2-4	**3**	0	1	2

Cameroon	1 (0.2%)	2 (1.5%)	2	**1**	0	1	0

LAM*	11 (2.6%)	4 (3%)	2	**2**	0	0	1

S	10 (2.4%)	4 (3%)	2	**2**	0	1	1

X	2 (0.5%)	2 (1.2%)	2	**1**	0	0	1

Bovis	2 (0.5%)	2 (1.5%)	2	**1**	0	0	1

Others	11 (2.6%)	0	n.a.				

Unclassified	183 (43.3%)	59 44.7%)	2-8	**19**	8	5	6

*Total*	423	*132*	*2-9*	***47***	*14*	*16*	*16*

*for one of the LAM clusters, country of birth of the cases is not known

n.a (not applicable)

Regarding the uninational clusters, there were a total of 14 clusters with German cases only and 4 immigrant clusters composed of cases from the same country. For one cluster information on country of birth was missing. In the mixed clusters we included three clusters with children born in Germany with migration background.

Among the MTBC genotypes that were identified in the study population, we looked in particular into the most frequent genotypes with the largest numbers of cases (Beijing and Haarlem) and we observed that the Beijing genotype was predominantly found in individuals born in the FSU with more than half of the cases belonging to clusters. The Haarlem genotype, instead, was mainly found in German and in East European cases with only one fifth of the cases being in clusters. Haarlem clusters were smaller (2 patients) than the Beijing ones (2 to 9 patients).

**Figure 2:**
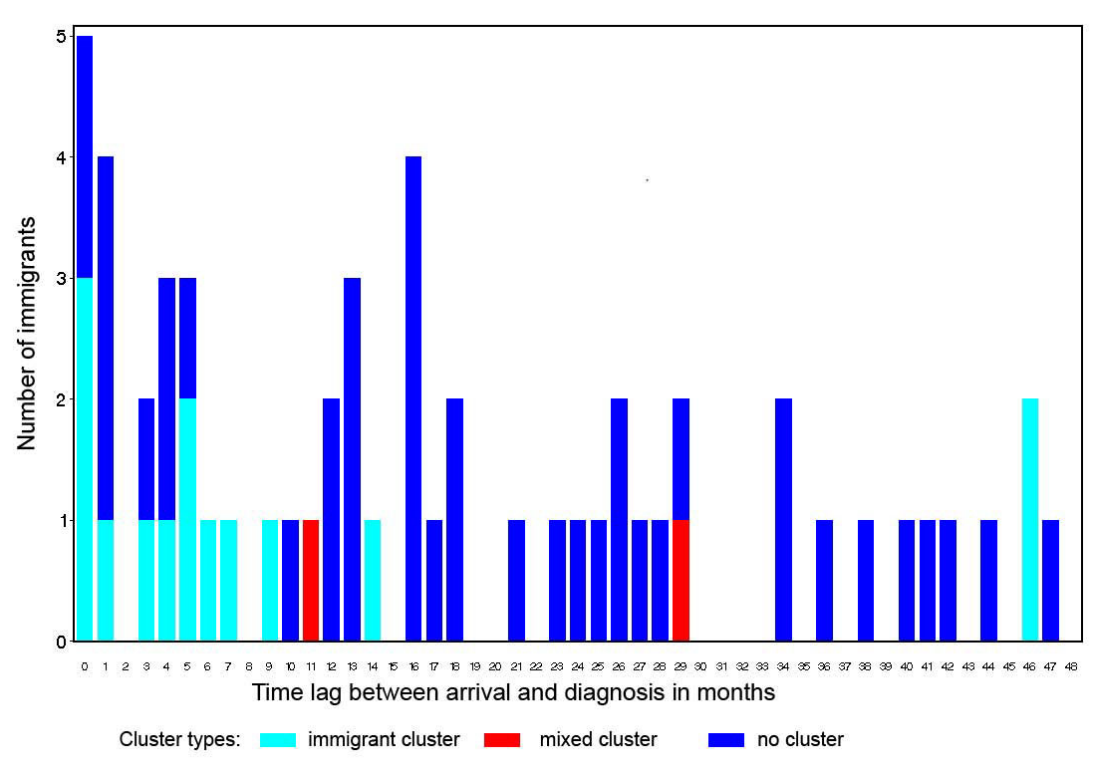
**Time lag between arrival in Germany and diagnosis**. Time between arrival in Germany and diagnosis in the immigrant sub-group.

To scrutinize the correlation of clusters over time after arrival in Germany we plotted the time lag between arrival and diagnosis of TB stratified by immigrants of mixed clusters, immigrant clusters and non-clustered cases where data was available. See Figure 2: Time between arrival in Germany and diagnosis in the immigrant sub-group.

Of the cases diagnosed within four years after arrival, 40 (71%) did not belong to a DNA fingerprint cluster. Two cases diagnosed within the first 48 months after arrival in Germany were found to belong to a mixed cluster: one from Nepal with a Delhi genotype diagnosed 11 months after arrival and one from Bosnia with a Haarlem genotype diagnosed 29 months after arrival in Germany. Fourteen cases were grouped in 6 immigrant clusters: 3 with Beijing genotype, 1 with Cameroon genotype and 2 unclassified. At a time span of one year between arrival and diagnosis, we find 24 cases, 12 in clusters and 12 non-clustered. Almost half of these cases originate from the FSU. Of interest is that 8 of the clustered cases had a Beijing genotype and all except one case came from the FSU. The other 4 clustered cases had Cameroon, East African Indian (EAI), Delhi and unclassified genotypes.

### Epidemiological links

For 13 (28%) of the 47 DNA fingerprint clusters, epidemiologic links between some members of the clusters could be identified. These 13 clusters include 32 individuals, for 26 of whom epidemiological links could be found. That is, for 19.7% of all clustered cases epidemiologic links were identified and confirmed. The majority of cases in these clusters (n = 10) had a definite epidemiologic link (members of a same family or household), followed by cases with a probable link (n = 3), namely people frequenting the same milieu and one of the cases was infected at work (health professional working with TB patients). For two clusters possible links between cluster members were suspected (homeless shelter), but could not be confirmed.

### Transmission risk

In our setting the proportion of German cases caused by foreign-born cases was estimated at 18.3% (95% CI: 8.3 - 28.3%). This calculation was based on 56 individuals whose date of diagnosis was within 12 months from another cluster member's. For this analysis we excluded those "mixed" clusters with immigrants and their children born in Germany. This is an overall estimate and it must be noted that the age of diagnosis for German cases in mixed clusters is lower than for Germans in German-only clusters (8.7 years lower in males and 11.6 years lower in females). This indicates that the above proportion decreases with age.

According to the TB case reports of Baden-Württemberg, over the years 2003 to 2005, 58% of all TB cases were foreign-born. If we assume a constant immigration of infected foreign-born persons and a random mixing between Germans and the foreign-born, we would also expect that 58% of all new infections in German cases are from a foreign-born person. Since our estimate is considerably lower, we concluded that there is no random mixing.

The estimated proportion of foreign cases caused by Germans is 16.0% (95% CI: 7.6 - 24.3%). This estimate is based on 74 foreign-born cases. This low percentage shows a high transmission of disease within foreigners, since, assuming random mixing, we would expect 100% - 58% = 42% cases caused by Germans.

### Degree of integration (Integration index)

The integration index was computed for the 217 immigrants with available questionnaires (corresponding to 57% of all the questionnaires received). For the correlation between integration index and time spent in Germany we used the year of arrival given in the study questionnaires, when this information was available (n = 207). Of these, 41 belonged to a DNA fingerprint cluster (26 to an immigrant-only cluster and 15 to a mixed cluster).

**Figure 3:**
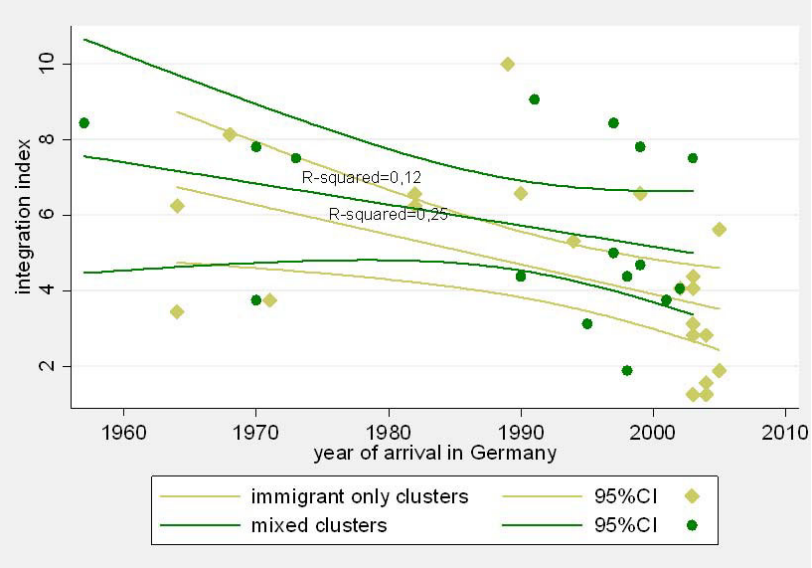
**Integration index vs. year of arrival**. Distribution of the integration index in a mixed cluster versus an immigrant-only cluster over time.

After omitting an outlier case who arrived in Germany in 1920, we observed that the mean crude integration index differed between the two groups of interest: immigrants in mixed clusters (mean: 4.57 (CI: 3.41 - 5.73) versus immigrants in immigrant-only clusters (mean 1.88: CI: 0.98 - 2.79). However, further linear regression analysis showed that this difference is not significant after adjustment for duration of residence. For illustration see Figure 3: Distribution of the integration index in a mixed cluster versus an immigrant-only cluster over time. Small numbers, however, do not allow further conclusions. Moreover, this analysis includes some immigrants in clusters (mixed or immigrant), which may in fact not be the result of recent transmission.

### Specific cluster types

We selected and interpreted (see discussion) some of the most representative clusters for each genotype:

1) Beijing genotype: cluster 3 is an immigrant cluster with individuals born in different FSU countries, where the Beijing genotype is known to be highly endemic. Cluster 35 is a mixed cluster with two immigrants and one German, with one of the immigrants and the German presenting the same Multi-Drug Resistance (MDR) pattern.

2) Cameroon genotype: cluster 9 is an immigrant cluster with one case from Togo and one case from Cameroon, where this genotype is endemic.

3) Delhi genotype: cluster 43 is a mixed cluster with an immigrant from Nepal (where this genotype is endemic) and a German. Cluster 15 is an epidemiologically confirmed "mixed" cluster with a Kenyan mother, her friend and her two children born in Germany. In contrast to cluster 15, there are no epidemiological links available to confirm cluster 43.

4) EAI genotype: cluster 5 is an immigrant cluster with a case from Romania and a case from the Philippines, and cluster 14 is an immigrant cluster with three Pakistanis. No contact tracing information is available for either of the clusters.

5) Haarlem genotype: cluster 12 and 45 are mixed clusters with a German and a Bosnian and a German and Yugoslavian (three countries where this genotype is endemic) with no epidemiological information available linking the members of these clusters. Cluster 8 is a German-only cluster formed by two individuals who had close contact, which has been confirmed through contact investigations.

6) LAM genotype: mixed cluster 6 is formed by a German and a Brazilian from a LAM endemic area.

7) Unclassified: cluster 26 was confirmed through extensive epidemiological contact investigations. The index case is a young female Algerian diagnosed in 2000 (outside the study period). A young Czech female contact was diagnosed end of 2002. In July 2003, molecular typing of a German contact showed an identical pattern with the index case. The third case diagnosed during the study period is an Iranian young male with no apparent connection to the others. The fourth case, diagnosed in April 2005, is a German female with contact to the index case. In December 2005, a young second generation Turkish man born in Germany was diagnosed. Seemingly, no contact exists between this case and the rest. For those with DST (Drug Sensitivity Test) information available, resistance patterns are identical.

For a full cluster description list see additional file 3: Cluster description table.

## Discussion

Tuberculosis is a global disease which travels in and between populations. Several studies have been carried out to address this problem. In our study we prospectively collected epidemiological and molecular data and data on social interaction between sub-groups from high- (immigrants) and low- (Germans) prevalence countries. To the best of our knowledge this it is the first time that someone has attempted to develop a tool to prospectively measure integration to better understand TB transmission between immigrants and the local population in a given country. The study questionnaire developed allowed us to collect not only data on social habits and interaction between groups, but also socio-economic and demographic data and data on risk behaviours associated with TB.

Of the 57.8% study participants born outside Germany, almost one third were German resettlers from the FSU and East Europe, followed by immigrants from Turkey and Sub-Saharan Africa. As in other studies, the well known phylogeographical distribution of mycobacterieal genotypes was observed in the study participants [30,24,23]. Cases from the FSU presented mainly strains of the Beijing lineage, while among the Germans and the East Europeans the Haarlem lineage was more common. The percentage of clustered cases was higher among patients of a Beijing genotype than patients of a Haarlem genotype.

With these findings it cannot fully be elucidated if the clustered cases indicate recent transmission in Germany or importation of strains that are endemic in particular home countries, i.e. a Beijing strain from the FSU. As in the case of Somalis in the Netherlands [3], Denmark [12] and London [31], reactivation of imported strains is likely to explain why clusters with cases from the FSU without any obvious epidemiological link occur in Germany. To determine whether transmission took place outside the country prior to immigration, in a reception centre after arrival or whether it is simply an endemic strain predominant in the area of origin depends on in depth epidemiological contact tracing investigations. There is an urgent need for enhanced international comparison of RFLP patterns to help confirming recent transmission.

Furthermore, when interpreting TB data we have to keep in mind that the risk of developing TB is greater in the first year after arrival in the host country [32] and that clustering of MTB in foreign-born persons increases with the duration of residence, which suggests that transmission of imported strains into the autochthonous population may occur after a lag time [33].

The analysis of the time lag between arrival in Germany and diagnosis shows that two thirds of the cases diagnosed within a year after arrival in Germany were from FSU and that half of them where diagnosed in the first month (Figure 2). This illustrates that screening programs for German resettlers in Germany [19,20] effectively target patients with imported TB belonging to this sub-group. Furthermore, the same analysis of the time lag between arrival in the host country and diagnosis confirmed that little transmission into the autochthonous population took place. Within 48 months between arrival in Germany and diagnosis of TB we only found two mixed clusters, one of which, as we will discuss later, may not even be the result of recent transmission.

A range of clusters for the most representative genotypes were selected to discuss (a) the problem of interpreting DNA fingerprint clusters as recent transmission versus being the result of imported endemic strains, (b) the significance of 'mixed clusters' for recent transmission between high-incidence populations (immigrants) and low-incidence populations (Germans), (c) the significance of endemic strains (e.g. Beijing, Haarlem) and (d) the concept of the integration index and its importance for understanding transmission dynamics in and between sub-groups:

1) The fact that most cases in Beijing immigrant cluster 3 were diagnosed within 1-6 months after arrival in Germany makes it very unlikely that transmission took place in one of the reception centres. We therefore assumed that this was a cluster indicating importation of a strain that is highly endemic in the FSU. However, recent transmission in Germany cannot be entirely ruled out, especially for those cases with the same DST pattern. Given the lack of epidemiological information from the contact investigations, this second hypothesis could not be proven. The same limitation applies to interpret cluster 35. This mixed cluster with two immigrants and one German case could represent recent transmission in Germany (see the identical DST pattern of the German and one of the immigrant isolates). While we observe comparatively low integration indexes in the immigrant cluster, the integration index in the mixed cluster is much higher.

2) Cameroon cluster 9 may be the result of recent transmission in Germany, with the case from Cameroon infecting the case from Togo. However, given the absence of epidemiological data linking the two cases this assumption could not be confirmed.

3) Although only one of the selected Delhi clusters was confirmed through contact investigations, we believe both clusters illustrate recent transmission in Germany: cluster 43 is a mixed cluster with an immigrant from a high-endemic country (Nepal) showing a high integration index and a German. Cluster 15 is an epidemiologically confirmed "mixed" cluster formed by a Kenyan mother, her two children born in Germany and a friend from Kenya visiting the family.

4) EAI cluster 5, with a Romanian and a Filipino, may represent recent transmission in Germany. Since no epidemiological information linking the two is available and this genotype is not endemic in either of the two countries, we reached the conclusion that both cases might have been infected by a case not captured in the cluster/study period. Cluster 14, instead, is an immigrant cluster formed by 3 cases from a highly EAI endemic country (Pakistan) with no apparent epidemiological link and thus pointing in the direction of an imported endemic strain.

5) As shown before, cluster 12 is a Haarlem mixed cluster with a German and a Bosnian, which, in the absence of epidemiologic information linking the two cases and the fact that the diagnosis dates are more than one year apart, we tend to interpret as casual contact of two cases from two countries where this genotype is endemic. The non-German case shows a relatively low integration index. Cluster 45 with a case from Germany and former Yugoslavia may also be an endemic cluster, since the DST patterns also differ.

6) LAM mixed cluster 6, with a German and a Brazilian, led us to the conclusion that since the LAM genotype is endemic in Brazil this cluster may be due to recent transmission in Germany. The Brazilian has been in Germany since 1957 and has a very high integration index.

As illustrated by the above examples, we observed a trend whereby most immigrants in mixed clusters showed higher integration indexes than immigrants in immigrant-only clusters, supporting our hypothesis that the higher the degree of integration in the host country the higher the likelihood to belong to a mixed cluster. This trend corroborates the strength of the tool in measuring integration among sub-groups. It is accepted that social marginalization, including associated factors such as alcohol abuse and nutritional deficiencies, pose a risk factor for TB [34]. We are sure that the investigation of integration patterns and its impact on TB transmission will be a helpful tool to design measures for TB prevention, control and treatment which are adapted and responsive to the needs of specific groups such as immigrants, but also other marginalized groups (i.e. homeless, drug addicts, etc.). With increasing numbers of studies in different countries and cities it has become clear that there is substantial setting specific variation in transmission patterns. A standardized tool for measuring integration patterns in relation to TB transmission might also be useful for comparison between countries.

In our study, the probability of cases among mixed clusters to be caused by a foreign-born case was 18.3%. This is much lower than expected under random transmission (58%). We can therefore conclude that integration is far from random with limited social interaction between foreigners and the autochthonous population. This rate includes two clusters (Haarlem cluster 12 and 46) which, as argued before, may not be a result of recent transmission. If this is true, the rate we give even overestimates the actual transmission risk meaning that the actual probability of cases to be caused by a foreign-born case would be even lower. The risk of transmission from immigrants to the local population from other published molecular studies vary between 17% in the Netherlands [3] to very low transmission rates in Denmark (0,9%) [12], Norway [7] or in cities like London [26], New York [2] or Hamburg (2.8%), where the study focused on a marginalized population.

If we look again at the mixed clusters we would like to note that of the 16 mixed clusters, apparently 7 were first identified in native-born Germans (35, 43, 12, 46, 31, 4 and 11). In cluster 43 and 31 the time difference between the identification of the German (supposedly index case) and the immigrant is less than one year, therefore it is still possible that the one with later diagnosis may have infected the earlier one. Mixed clusters with a German index case support, once again, our conclusion that the effect of imported TB on transmission into the autochthonous population is lower than expected.

Cluster 5, 24, 22, 41, 20 and 27 suggest that recent transmission amongst immigrant sub-groups is taking place in Germany. This is certainly a problem that deserves further research. However, our study sub-population sizes were not sufficient to carry out further stratification.

As most studies, our study may underestimate recent transmission due to the limited time of observation. The full size of the clusters is not captured due to the fact that TB transmission chains are building up slowly and delays in diagnosis can be substantial. One recent study in Norway analyzed retrospectively the transmission dynamics of TB between sub-groups over an extended period of time [7]. They found that in twelve years, imported TB by immigrants from high-incidence countries had little influence on the transmission into the receiving low-incidence country. On average per year, only 2 non-immigrants and 13 immigrants developed disease as a result of transmission within the country by imported TB.

A limitation of our study was that only half of the study participants completed the study questionnaire. This may be in part explained by the situation of the foreign-born study participants (mobility of the immigrants, language barriers, fear of legal consequences, etc.). Translating the questionnaire into eleven languages could not overcome the language barrier. The crucial issue appears to be the capacity of the local and state health offices to support the data collection at the scale needed for a complete coverage.

## Conclusion

In order to describe TB transmission dynamics between high- and low- endemic populations it is necessary to be able to distinguish between recent transmission and activation of imported endemic strains. Molecular typing is a valuable contribution to epidemiological analysis provided it is combined with thorough epidemiological investigations. As our study results reveal, contact investigations would need further improvement. Having this in mind, the results obtained in this study confirm the findings of other studies that TB transmission from TB high-prevalence immigrant populations to TB low-prevalence local populations is lower than one would expect under random transmission. This may be explained by the good performance of TB screening programmes at entry for certain migrant groups arriving in the host country from high-prevalence countries, by a low degree of integration between immigrants and the local population or by a combination of both.

Measuring the integration of immigrants into the local social environment showed a trend supporting the hypothesis that immigrants in mixed clusters had higher integration indexes than those in immigrant clusters. Furthermore, we are convinced that this tool has great potential to improve control by measuring the degree of integration and its impact on TB transmission in settings with low-prevalence TB in the local population and immigration from high-incidence countries.

One goal of this investigation at the local level of the public health offices was achieved: the preparation and participation in this study effectively refined contact tracing instruments in Baden-Württemberg. This study demonstrates that a combination of conventional contact tracing in high risk groups with the input of molecular typing data improves targeted case management of tuberculosis in heterogeneous populations.

## Competing interests

The authors declare that they have no competing interests.

## Authors' contributions

JB participated in the data collection and analysis, study coordination and writing of the manuscript. SN carried out the DNA fingerprinting, Genotype analysis, Cluster analysis and helped to draft the manuscript. VRL performed statistical analysis and helped to draft the manuscript. BB participated in data collection and gave input in the drafting of the manuscript. CD participated in the design of the study/study proposal, data collection and gave input in the drafting of the manuscript. ER participated in data collection and gave input in the drafting of the manuscript. HB contributed to the study design, data analysis and drafting of the manuscript. WH participated in the design of the study/study proposal, carried out data analysis and helped to draft the manuscript. TJ participated in the study design/study proposal and coordination, analysis of data and writing of the manuscript. All co-authors read and approved the final manuscript.

## Pre-publication history

The pre-publication history for this paper can be accessed here:

http://www.biomedcentral.com/1471-2334/9/197/prepub

## Supplementary Material

Additional file 1 Study questionnaire in English Click here for file

Additional file 2 Questions and scoring used for the computation of the integration index Click here for file

Additional file 3 Cluster description table Click here for file
