# Supplementary material for: Transmission dynamics of pulmonary tuberculosis between autochthonous and immigrant sub-populations
Source: BMC Infect Dis. 2009 Dec 4;9:197. doi: 10.1186/1471-2334-9-197 (PMC3224697; doi:10.1186/1471-2334-9-197)
Supplement: Additional file 1 — Study questionnaire in English [file 1471-2334-9-197-S1.PDF]

I Englisch/English

Laufende Nr.
für I: |_|_|

Universitätsklinikum Heidelberg
Abteilung Tropenhygiene und öffentliches Gesundheitswesen

MuT-*Studie*

Migration und Tuberkulose in Baden-Württemberg

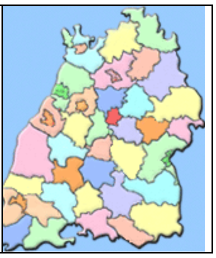

Fragebogen Baden-Württemberg für Indexpersonen

Dieser Fragebogen wird von den Patienten selbst u.U. mit Hilfe eines/-r Mitarbeiters/-in des Gesundheitsamts ausgefüllt.

Vom Gesundheitsamt auszufüllen:

Gesundheitsamt: ____________________________

GA-Kennung (Octoware): 1.08._.__.

MeldungsNr. (Octoware) der befragten Indexperson:

[_ _ _ _ _ _ _ _ _ _ _ _ _]

MeldungsNr. (Octoware) falls im GA bekannt [_ _ _ _ _ _ _ _ _ _ _ _ _]
des in Frage 26 genannten „bekannten Kontaktes zu einem Tuberkulosepatienten“

Datum der Befragung (Tag/Monat): __/__/200_

MuT-Studie: Migration und Tuberkulose in Baden-Württemberg

(1) What is your month and year of birth? |_|_| / |_|_|_|_|
month year

(2) What is your sex?

[] female
[] male

(3) What is the zipcode of your town of residence? |_|_|_|_|_|

(4) Where do you live? *(tick one!)*

[] in your own flat /apartment/ house → ***Please go to question number 5!***
[] no permanent address
[] in communal accommodation
[] in an old people's/nursing home
[] in a place of detention
[] other:..
[] unknown / no information available

} ***Please go to question number 7!***

(5) How many rooms does your flat have (without kitchen and bathroom)?

Give number in box: [___]

(6) How many people live in your flat – including yourself?

Give number in box: [___]

(7) Where do people you are living with come from?
(What are their countries of origin?)

____

____

____

(8) At home, how many people have slept in the same room as you lately?

[] you alone
[] 2-5 persons
[] more than 5 persons

(9) Where do your closest neighbours come from?
(What are their countries of origin?)

____

____

____

(10) Do you currently go to regular school or vocational school?
[] yes
[] no
[] unknown / no information available

(11) Do you currently go to university or polytech?
[] yes
[] no
[] unknown / no information available

(12) Do you currently attend any other classes?
[] yes
[] no
[] unknown / no information available

(13) What did you train for? ....

(14) Are you currently employed?
[] yes
[] no → ***Please go to question number 18!***
[] unknown / no information available

(15) For how many years have you been in this job?
[] for less than 1 year
[] for 1-5 years
[] for more than 5 years
[] unknown / no information available

(16) Describe your present job? ....

(17) In which sector/trade/industry do you work? ....

(18) With whom do you spend your time at work/ at school
(What are their countries of origin?)

____

____

____

[] I´m unemployed / I don´t go to school at present

(19) Do you spend much of your spare time with people –other than your family?
[] yes, almost daily
[] yes, from time to time
[] seldom or never

(20) Do you go to a place of worship (e.g. church, mosque...) where you meet people of your religion?

[] never
[] seldom
[] once a week
[] twice/three times a week
[] more often
[] unknown / no information available

(21) Do you go to clubs/pubs etc.?

[] never
[] seldom
[] once a week
[] twice/three times a week
[] more often
[] unknown / no information available

(22) Where do the people you meet in your spare time come from?
(What are their countries of origin?)

____

____

____

(23) Do you have a warm meal regularly (i.e. more than 4 times a week)

[] yes
[] no

(24) Do you smoke?

[] yes
[] no

(25) Do you drink alcohol every day?

[] yes
[] no

(26) Have you had contact with a known tuberculosis patient within the last 3 years?

[] No / unknown ——→ ***Please go to question number 29!***
[] Yes

(27) What was the country of origin of this tuberculosis patient?.....................................

(28) In which country did the contact take place? ....

(29) What is:

(29a) your country of birth? ....

(29b) your nationality/citizenship? / Which passport do you have?

(Multiple answers possible!)..

(29c) your ethnic origin? ....

(29d) the country of birth of your mother? ....

(29e) the country of birth of your father? ....

(29f) the country where you lived longest before you came to Germany? ....

(30) Please give us the month and year of your first entry to Germany:

|_|_| / |_|_|_|_|
month year

[] unknown / no information available

(31) Have you visited your country of origin, or that of your parents, since then?

[] no → ***Please go to number 33!***
[] my country of origin and that of my parents is Germany>***Please go to number 33!***
[] yes
[] unknown / no information available

(32) Please tell us the entire duration of your stay there for the past 3 years:
Give us the number in the small box:

2003 [___] weeks

2002 [___] weeks

2001 [___] weeks

2000 [___] weeks

[] my last visit was before 2000

(33) Do you have close relatives not living in Germany?

[] no
[] yes, in particular: *(Multiple answers possible!)*
- [] mother
- [] father
- [] wife/ husband
- [] children under 15
- [] children over 15
- [] sisters / brothers
- [] unknown / no information available

(34) Which language do you mainly speak here in Germany?
[] German
[] other languages

(35) Do you read German newspapers?
[] never
[] sometimes
[] regularly

(36) How do you judge your knowledge of **German**?

Speaking:	Writing:
[] fluent	[] fluent
[] good	[] good
[] basic	[] basic
[] poor	[] poor
[] I cannot speak it at all	[] I cannot write it at all

(37) How do you judge your knowledge of of **your mother tongue** (if not German?

Speaking:	Writing:
[] fluent	[] fluent
[] good	[] good
[] basic	[] basic
[] poor	[] poor
[] I cannot speak it at all	[] I cannot write it at all

Thank you very much for your cooperation
