# Supplementary material for: Transmission dynamics of pulmonary tuberculosis between autochthonous and immigrant sub-populations
Source: BMC Infect Dis. 2009 Dec 4;9:197. doi: 10.1186/1471-2334-9-197 (PMC3224697; doi:10.1186/1471-2334-9-197)
Supplement: Additional file 2 — Questions and scoring used for the computation of the integration index [file 1471-2334-9-197-S2.DOC]

### Questions and scoring used for the computation of the integration index

| Questions | | Answer categories | Score |
| --- | --- | --- | --- |
| Q04: | Where do you live? | Own appartment | +1 |
|  |  | No permanent address | -0.5 |
|  |  | Communal accomodation | -1 |
|  |  | Nursing home | +0,5 |
|  |  | Detention centre/prison | -0,5 |
| Q07: | Where do the people living with you come from? | German only | +1 |
|  |  | Foreign only | -1 |
|  |  | Mixed | +0.5 |
| Q10: | Do you currently attend a school? |  |  |
| Q11: | Do you currently attend University? | Yes/No (questions pooled) | If yes for one of them= +1 |
| Q12: | Do you attend some kind of training? |  | If no to all of them = -1 |
| Q14: | Do you have a permanent employment? |  |  |
| Q18: | Where do your colleagues come from? | German only | +1 |
|  |  | Foreign only | -1 |
|  |  | Mixed | +0.5 |
| Q22: | Where do your friends come from? | German only | +1 |
|  |  | Foreign only | -1 |
|  |  | Mixed | +0.5 |
| Q34: | What language do you speak in Germany? | mostly German | +1 |
|  |  | mostly not German | -1 |
| Q35: | Do you read German newspapers | often | 1 |
|  |  | sometimes | +0,5 |
|  |  | never | -1 |
| Q36: | How good is your German? | very good | +1 |
|  |  | good | +1 |
|  |  | relatively good | +0.5 |
|  |  | rather poor | -0.5 |
|  |  | I don’t speak at all | -1 |
