# Supplementary material for: Transmission dynamics of pulmonary tuberculosis between autochthonous and immigrant sub-populations
Source: BMC Infect Dis. 2009 Dec 4;9:197. doi: 10.1186/1471-2334-9-197 (PMC3224697; doi:10.1186/1471-2334-9-197)
Supplement: Additional file 3 — Cluster description table [file 1471-2334-9-197-S3.DOC]

G: German case M: Immigrant/Migrant case; Epi links: Clusters with epidemiologically confirmed links

I: Integration index; n.a. not available; TO ALL (drugs tested for sensitivity: INH, RMP, PZA, EMB and SM)
